# A Nurses' Club for Dublin

**Published:** 1919-09-20

**Authors:** 


### A Nurses' Club for Dublin.

The College of Nursing in Ireland is embarking on a most commendable enterprise, the establishment of a Club for Nurses. Sir John Lumsden, K.B.E., is acting as Chairman of the promoting Committee, which is endeavouring to raise between £1,000 and £1,500 for the purpose. I have not at the moment the names of all the members, but there are a few well-known women associated with the movement, including Mrs. Hignett, O.B.E., and Miss Macnie, both of whom have wrought miracles of achievement in raising money for the Red Cross.

### Design of the Club.

The Club is designed to accommodate all trained nurses, whether they are College members or not. There will probably be a dozen beds, a library, reading- and writing-rooms, and, in a word, every accommodation that a modern well-equipped club should contain. And, needless to observe, the scale of charges will be well within the means even of the ill-paid Irish nurse. If the establishment is efficiently conducted—and there is no reason to doubt that it will—there is bound to be an early demand for its extension. All Irish nurses who travel anywhere usually find it necessary to come to Dublin. For these the Club will be a great boon. But it will confer a much greater benefit on the Dublin nurses by providing a place where they can turn to when off duty to enjoy a rest, a little privacy, or a cup of tea with their friends.

### The Club Membership.

Although the College of Nursing is providing this Club, membership thereof will be open to all, and I am glad to note that the Committee are determined that it shall be free from sectarian bias. Its complete and ultimate success will depend absolutely and altogether on the members themselves, and I would suggest that it should be opened with a stirring heart-to-heart appeal for comradeship and *esprit de corps*. Let there be a complete absence of snobbishness, of cliques, and of political and sectarian bias. In such conditions the Club will boom.

